# A unified model of post-stroke language deficits including discourse production and their neural correlates

**DOI:** 10.1093/brain/awaa074

**Published:** 2020-04-24

**Authors:** Reem S W Alyahya, Ajay D Halai, Paul Conroy, Matthew A Lambon Ralph

**Affiliations:** a1 MRC Cognition and Brain Sciences Unit, University of Cambridge, Cambridge, UK; a2 King Fahad Medical City, Riyadh, Saudi Arabia; a3 Division of Neuroscience and Experimental Psychology, University of Manchester, Manchester, UK

**Keywords:** stroke, aphasia dimensions, connected speech production, discourse, lesion-symptom mapping

## Abstract

The clinical profiles of individuals with post-stroke aphasia demonstrate considerable variation in the presentation of symptoms. Recent aphasiological studies have attempted to account for this individual variability using a multivariate data-driven approach (principal component analysis) on an extensive neuropsychological and aphasiological battery, to identify fundamental domains of post-stroke aphasia. These domains mainly reflect phonology, semantics and fluency; however, these studies did not account for variability in response to different forms of connected speech, i.e. discourse genres. In the current study, we initially examined differences in the quantity, diversity and informativeness between three different discourse genres, including a simple descriptive genre and two naturalistic forms of connected speech (storytelling narrative, and procedural discourse). Subsequently, we provided the first quantitative investigation on the multidimensionality of connected speech production at both behavioural and neural levels. Connected speech samples across descriptive, narrative, and procedural discourse genres were collected from 46 patients with chronic post-stroke aphasia and 20 neurotypical adults. Content analyses conducted on all connected speech samples indicated that performance differed across discourse genres and between groups. Specifically, storytelling narratives provided higher quantities of content words and lexical diversity compared to composite picture description and procedural discourse. The analyses further revealed that, relative to neurotypical adults, patients with aphasia, both fluent and non-fluent, showed reduction in the quantity of verbal production, lexical diversity, and informativeness across all discourses. Given the differences across the discourses, we submitted the connected speech metrics to principal component analysis alongside an extensive neuropsychological/aphasiological battery that assesses a wide range of language and cognitive skills. In contrast to previous research, three unique orthogonal connected speech components were extracted in a unified model, reflecting verbal quantity, verbal quality, and motor speech, alongside four core language and cognitive components: phonological production, semantic processing, phonological recognition, and executive functions. Voxel-wise lesion-symptom mapping using these components provided evidence on the involvement of widespread cortical regions and their white matter connections. Specifically, left frontal regions and their underlying white matter tracts corresponding to the frontal aslant tract and the anterior segment of the arcuate fasciculus were particularly engaged with the quantity and quality of fluent connected speech production while controlling for other co-factors. The neural correlates associated with the other language domains align with existing models on the ventral and dorsal pathways for language processing.

## Teaser

Alyahya *et al.* propose a unified model of post-stroke language deficits that captures the multidimensionality of connected speech production, explored using various discourse genres in patients with post-stroke aphasia and in neurotypical controls, and identify the neural correlates of core language dimensions.

## Introduction

Connected speech production is a complex task, which involves several processes related to conceptual and semantic preparation, lexical access, syntactic and phonological encoding, and motor execution of the words by the articulatory system (Garrett, 1984; Dell *et al.*, 1997; Levelt *et al.*, 1999). Patients with acquired language impairments (i.e. aphasia) secondary to brain damage or neurological disorders have multiple underlying impairments, including deficits in connected speech production, and it is not, perhaps, surprising that their fluency is usually compromised. For differential diagnosis and clinical management purposes, a common but very simplistic approach has been taken to fluency, which reduces it to a single dimension/dichotomy, in which patients are classified as being ‘fluent’ versus ‘non-fluent’ capture by classical measures on standardized tests, such as the Boston Diagnostic Aphasia Examination (BDAE) (Goodglass and Kaplan, 1983) or the Western Aphasia Battery (WAB) (Kertesz, 1982). Measuring fluency and connected speech production is important for defining therapy objectives, including duration and intensity of training; charting intervention effectiveness; and monitoring patient’s progress. Fluency assessment, however, is complicated by which elicitation tasks to use and which measures to extract. If connected speech is a multifaceted construct then it is important for assessments to be based on rich data, to extract multiple measures and to check that the results are consistent across different elicitation techniques, including the commonly used composite picture description alongside more naturalistic forms of connected speech production, such as storytelling narratives and procedural discourse. Although those naturalistic forms can provide rich samples, they are often long in duration and require time-consuming quantitative coding, and thus are less utilized in both clinical and research settings. It has been argued that the validity of fluency in aphasia is not well defined as a result of the heterogeneity in the literature (Park *et al.*, 2011). Few studies have compared connected speech measures across different elicitation techniques. No differences were found between picture-supported and unsupported storytelling narratives in aphasia in measures of discourse content, productivity and complexity (Doyle *et al.*, 1998). Conversely, storytelling was found to probe more quantity and diversity than picture description in aphasia and neurotypical adults (Fergadiotis and Wright, 2011), and when comparing fluent to non-fluent aphasia (Wright *et al.*, 2003). 

Only a handful of previous studies have explored the lesion correlates of fluency and in all cases only a single picture description task has been used. For instance, reduced fluency in post-stroke aphasia has been associated with damage in the left inferior frontal gyrus and the left precentral gyrus (Borovsky *et al.*, 2007; Halai *et al.*, 2017). Other studies have also identified the anterior insula and anterior temporal areas (Borovsky *et al.*, 2007), and white matter tracts, corresponding to the anterior segment of the arcuate fasciculus (Fridriksson *et al.*, 2013; Basilakos *et al.*, 2014) and the frontal aslant tract (Catani *et al.*, 2013; Halai *et al.*, 2017) to be damaged in post-stroke aphasia, apraxia of speech, and primary progressive aphasia. This needs to be expanded to reflect the potentially more complex nature of connected speech production.

In this study, we identified the fundamental components of connected speech production within the context of other core language and cognitive domains in a unified model. Multiple research groups have achieved this aim by using multivariate decomposition algorithms: principal component analysis (PCA) (Kümmerer *et al.*, 2013; Butler *et al.*, 2014; Mirman *et al.*, 2015; Feenaughty *et al.*, 2017; Halai *et al.*, 2017; Lacey *et al.*, 2017; Alyahya *et al.*, 2018*b*). This data-driven approach maximizes the amount of shared variance in a heterogeneous sample and accounts for systematic variations across tests. By applying a varimax rotation to the PCA model, independent (orthogonal) components are generated with the added constraint that individual tests load maximally on one component and minimally on others. Previous studies have found consistent components related to phonology and semantics (Kümmerer *et al.*, 2013; Butler *et al.*, 2014; Mirman *et al.*, 2015; Lacey *et al.*, 2017; Alyahya *et al.*, 2018*b*), in addition to one component for fluency (Feenaughty *et al.*, 2017; Halai *et al.*, 2017; Lacey *et al.*, 2017). However, in the latter studies, only one elicitation technique (picture description or spontaneous speech) was used. Indeed, if connected speech reflects multi-dimensional constructs (Gordon, 1998) then this could be under-represented in the PCA outcomes if only one technique that elicits one type of discourse genre is used. Isolating the components of connected speech and identifying their neural correlates allows for a much more nuanced approach to clinical rehabilitation for patients with aphasia, in order to guide differential diagnosis and shape therapy objectives to help improve expressive deficits.

The current study addresses three main issues. First, to compare the quantity, diversity and informativeness of connected speech responses across three different discourse genres: a relatively simple and commonly used composite picture description task, and two more naturalistic forms of production elicited with and without picture-supported stimuli (storytelling narrative, and procedural discourse, respectively). Personal discourse, such as ‘tell me about your stroke’ was not used, as these samples are likely to be heterogeneous and rely heavily on long-term memory. To examine the performance for patients with aphasia as a group, the comparison was initially made between age/education-matched neurotypical adults and patients with aphasia. This was followed by a comparison between the fluent and non-fluent aphasia patients. The second aim was to provide a unified model of post-stroke language deficits while exploring the multi-dimensionality of connected speech within the wider context of neuropsychological/aphasiological deficits. This allows for a detailed examination of the interactions between multiple language processes while exploring the core components of connected speech production. The final aim was to map the fundamental components of post-stroke language deficits onto the brain lesions by performing whole-brain voxel-wise lesion-symptom mapping.

## Materials and methods

### Participants

Forty-six patients who had developed aphasia following a single left haemorrhagic or ischaemic stroke were tested in the chronic stage (>12 months post-stroke). Aphasia was diagnosed and classified using the BDAE (Goodglass and Kaplan, 1983). Participants were native English speakers with normal or corrected-to-normal vision and/or hearing. The exclusion criteria included multiple strokes or any other neurological conditions, severe motor-speech disorders, visual neglect, any contraindications for MRI scanning, being pre-morbidly left-handed, and patients who did not produce any response in all discourse samples. No restrictions were placed according to aphasia severity or classification, in order to sample the full range of aphasia symptoms. In addition to the patients, discourse samples were collected from 20 age- and education-matched neurotypical adults. All participants were native English-speakers, right-handed, and reported no abnormal neurological conditions or history of brain injury. Demographic information of both groups is presented in Table 1. Informed consent was obtained from all participants prior to participation under approval from local ethics committee.

**Table 1:** Participants’ demographic information

Demographic variables	Neurotypical adult group (*n *=* *20)	Aphasia group (*n *=* *46)
Gender, male:female ratio	9:11	32:14
Age, mean (range, SD)	68.85 (57–84, 8.47)	63.21 (44–87, 11.93)
Education, mean (range, SD)	14 (9–19, 2.8)	12.65 (9–19, 2.59)
Time-post stroke onset: mean months (range, SD)	N/A	69.43 (16–280, 48.86)
Lesion volume, voxel mm^3^, mean (range, SD)	N/A	15 497 (175–41 379, 11 188)
BDAE aphasia classification	N/A	Fluent aphasia
		Anomia = 20
		Conduction = 4
		Transcortical sensory = 1
		Non-fluent aphasia
		Transcortical mixed = 1
		Broca’s = 9
		Mixed non-fluent = 8
		Global = 3

BDAE = Boston Diagnostic Aphasia Examination; N/A = not applicable; SD = standard deviation.

### Discourse samples

Three discourse samples were collected from each participant with no time limit: (i) descriptive discourse, elicited using the ‘Cookie Theft’ composite picture from the BDAE (Goodglass and Kaplan, 1983); (ii) narrative discourse, elicited using a ‘Dinner Party’ storytelling script (Fletcher and Birt, 1983), which involved a series of eight black-and-white sequences of pictures; and (iii) unsupported procedural discourse, which was elicited by asking participants to describe ‘how they prepare a cup of tea’. No prompts or questions were provided except for non-verbal encouragement. In the first two discourses, participants were presented with the picture stimuli and were asked to look through them before producing a response.

Each sample was digitally recorded, transcribed verbatim (orthographically) and then used for content analyses by R.S.W.A. Four measures were extracted from each sample. First, the number of content words was counted as a measure of quantity of production. Content word count is one of the most widely used measures in fluency research and has been shown to have good validity (Feyereisen *et al.*, 1991). This measure includes words that are intelligible, informative and relevant to the content (adapted from Nicholas and Brookshire, 1993), including nouns, verbs, adjectives, adverbs, pronouns, conjunctions, articles, prepositions, numerals, and possessives; but excluding immediate repetition or perseverations of the same word or utterance. Contractions (e.g. it’s or haven’t) were counted as two separate words. Second, the number of distinct content words (type count) was coded as a measure of lexical diversity. This measure was selected because it is less sensitive to sample length, which can vary between participants and across different discourse genres. Third, informativeness was coded as a measure of quality in terms of information accuracy and appropriateness. This was computed by dividing the total number of content words by the total number of tokens and converting this into percentage. Finally, words per minute (WPM) measuring speech rate was obtained by dividing the total number of tokens by the duration of the sample.

### Background neuropsychological assessments

An extensive neuropsychological/aphasiological battery that assesses language and cognitive abilities was used (Butler *et al.*, 2014; Alyahya *et al.*, 2018*b*, 2020). This consisted of: (i) naming tests, including the Boston Naming Test (BNT) (Kaplan *et al.*, 1983), the Object and Action Naming Battery (OANB) (Druks and Masterson, 2000), and the 64-item Cambridge naming test (Bozeat *et al.*, 2000); (ii) comprehension tests, including noun and verb picture-to-word matching (Alyahya *et al.*, 2018*b*), 96-synonym judgement test (Jefferies *et al.*, 2009), verb synonym judgement test (Alyahya *et al.*, 2018*a*), and spoken sentence comprehension task from the Comprehensive Aphasia Test (Swinburn *et al.*, 2005); (iii) tests from the Cambridge Semantic Battery (Bozeat *et al.*, 2000): spoken word-to-picture matching, written word-to-picture matching, and a picture version of the Camel and Cactus test; (iv) subtests from the Psycholinguistic Assessments of Language Processing in Aphasia (PALPA) (Kay *et al.*, 1992): auditory discrimination using non-word (PALPA 1) and word minimal pairs (PALPA 2), and immediate and delayed repetition of non-words (PALPA 8) and words (PALPA 9); and (v) cognitive tests including: the Brixton Spatial Rule Anticipation Task (Burgess and Shallice, 1997), forward and backward digit span (Wechsler, 1987), and Raven’s Coloured Progressive Matrices (Raven, 1962).

### Acquisition and processing of neuroimaging data

**Figure 1:**
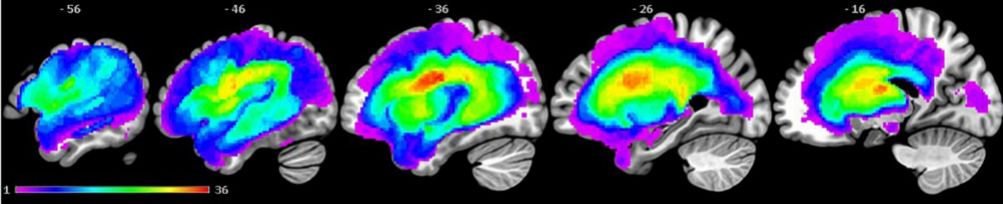
**Lesion overlap map across 46 post-stroke aphasia patients illustrating the distribution of lesions.** Colour scale indicates number of patients with a lesion at that location. The greatest lesion overlap among the patients (*n *=* *36) was in the left central opercular cortex (MNI coordinate: −38, −9, 24).

High-resolution structural T_1_-weighted MRI scans were acquired for each patient on a 3.0 T Philips Achieva scanner (Philips Healthcare) using an eight-element SENSE head coil. A T_1_-weighted inversion recovery sequence with 3D acquisition was used. Parameters are listed in the Supplementary material. Participants’ structural T_1_-weighted MRI scans were preprocessed with Statistical Parametric Mapping software (SPM8: Wellcome Trust Centre for Neuroimaging, http://www.fil.ion.ucl.ac.uk/spm/) running under MATLAB (version 2012a). Preprocessing details are explained in previous studies as the same participants were tested (Alyahya *et al.*, 2018*a*, *b*, 2020) and for completeness in the current paper, the details are provided in the Supplementary material. Figure 1 shows a lesion overlap map.

### Statistical analyses

#### Differences between discourse genres

We conducted four 2 × 3 mixed ANOVAs for each connected speech measure, with group (neurotypical versus patients with aphasia) as the between-subject factor and discourse genre (composite picture description versus storytelling narrative versus procedural) as the within-subject factor. The dependant variable for the ANOVAs were: (i) number of content words (quantity); (ii) type count (lexical diversity); (iii) informativeness (quality); and (iv) WPM (speech rate), with an alpha of *P *=* *0.0125 (Bonferroni corrected for multiple comparison). All significant interactions were explored using *post hoc t*-tests, and Bonferroni corrected for multiple comparisons. These analyses were repeated, but instead, the between-subject group factor involved fluent (*n *=* *25) versus non-fluent (*n *=* *21) aphasia groups, the patients were grouped according to their aphasia classification as diagnosed using the BDAE and demonstrated in Table 1.

#### A unified model of post-stroke language deficits

Patients’ scores on the connected speech measures from the three discourse samples were entered into a PCA along with their scores on all background neuropsychological tests. Components with an eigenvalue > 1.0 were extracted and then varimax rotated, which maximizes the loading of a measure onto one component for easier interpretations of the final solution. To validate the stability of the PCA solution, a *k*-fold cross-validation approach was used (*k* = 4), which determines which N-component solution provides the best prediction for a left out set of data (using root mean squared error) over 1000 permutations (Ballabio, 2015). The sample size for this PCA was adequate with a Kaiser-Meyer-Olkin index of 0.73, and Bartlett’s test of sphericity was significant (χ^2^ = 2087.9, *P *<* *0.001). This indicates that the sample had sufficient collinearity within the dataset.

#### Analysis of neuroimaging data

The unified PCA model was mapped onto brain lesions using a voxel-based correlational methodology (VBCM) (Tyler *et al.*, 2005). In contrast to voxel-lesion symptom mapping (Bates *et al.*, 2003), which uses binary lesion status of a voxel, VBCM identifies statistical relationships between brain and behavioural by correlating the signal intensity per voxel (as a continuous value) with the behavioural performance (see Geva *et al.*, 2012 for a comparison between the two methods). A multiple regression model was created using the normalized-smoothed T_1_-weighted images as the dependant variable and language and cognitive component as regressors of interest, entered simultaneously along with the other components and demographic variables (age, education, and time since stroke onset). Two parallel analyses were performed, with/without lesion volume as a covariate, to avoid a possible risk for type II error with the inclusion of lesion volume. The results were thresholded at *P *<* *0.001 voxel-level and cluster-level corrected using family-wise error (FWE) at *P *<* *0.05.

### Data availability

The conditions of our ethical approval do not permit public archiving of anonymized patient data. All data necessary for reproducing the results in this article can be requested from the senior author.

## Results

### Differences between discourse genres

#### Neurotypical subjects and patients with aphasia

**Figure 2:**
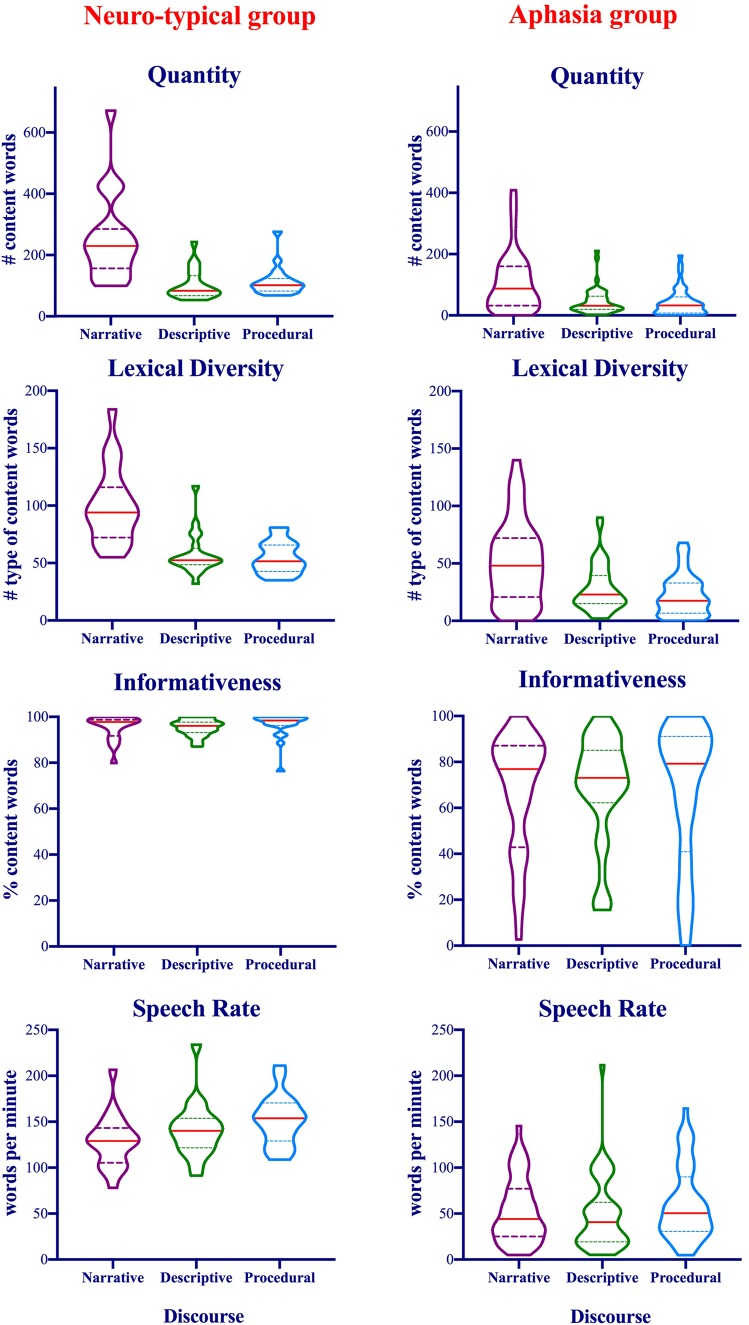
**Violin plots showing the distribution of the data and the probability density of four connected speech measures produced during three discourse genres among both neurotypical and patients with aphasia groups.** Straight red lines refer to the group median, *top* dotted lines refer to the third quartile, and *bottom* dotted lines refer to the first quartile.

The distribution of the data is illustrated in Fig. 2. The ANOVA results revealed significant group effects with better performance among the neurotypical group compared to the patient group on all measures: quantity of production [*F*(1,64) = 31.54, *P *<* *0.001, partial η^2^ = 0.33], lexical diversity [*F*(1,64) = 40.69 *P *<* *0.001, partial η^2^ = 0.39], informativeness [*F*(1,64) = 26.9, *P *<* *0.001, partial η^2^ = 0.3], and speech rate [*F*(1,64) = 95.97, *P *<* *0.001, partial η^2^ = 0.6]. The results revealed significant main effects of discourse genre for the quantity of production [*F*(2,128) = 73.86, *P *<* *0.001, partial η^2^ = 0.54], lexical diversity [*F*(2,128) = 96.67, *P *<* *0.001, partial η^2^ = 0.6] and speech rate [*F*(2,128) = 12.02, *P *<* *0.001, partial η^2^ = 0.15]. For quantity of production, storytelling narrative was significantly higher than both composite picture description and procedural discourse (*P *<* *0.001). For lexical diversity, storytelling was again the highest, followed by composite picture description and then procedural discourse. The differences between storytelling and the other two discourses were significant (*P *<* *0.001), and the differences between picture description and procedural discourse was also significant (*P *=* *0.01). Finally, for speech rate, the fastest speech was produced during procedural discourse, followed by picture description, and then storytelling. The differences, however, were only significant between procedural discourse and storytelling (*P *<* *0.001), and procedural discourse and picture description (*P *=* *0.002).

**Figure 3:**
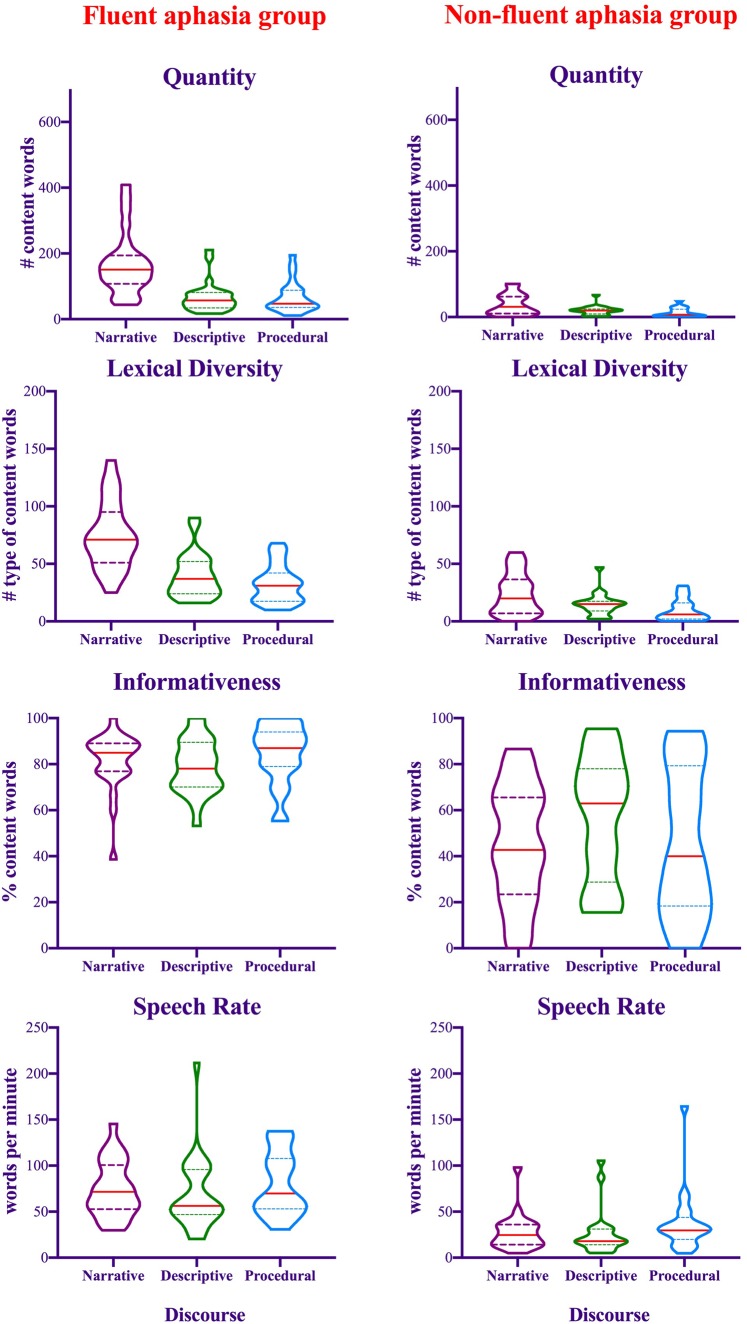
**Violin plots showing the distribution of the data and the probability density of four connected speech measures produced during three discourse genres among both fluent and non-fluent aphasia groups.** Straight red lines refer to the group median, *top* dotted lines refer to the third quartile, and *bottom* dotted lines refer to the first quartile.

The Group × Discourse interaction effects were significant for quantity of production [*F*(2,128) = 10.91, *P *<* *0.001, partial η^2^ = 0.15], and lexical diversity [*F*(2,128) = 6.14, *P *=* *0.003, partial η^2^ = 0.087]. Both interactions were driven by significantly higher quantity and diversity produced during storytelling narrative compared to the other two discourses for both groups (*P *<* *0.001). The differences between picture description and procedural discourse were significant for diversity among the patient group only, in favour of picture description (*P *=* *0.002). The differences between the two groups were significant for all three discourses with better performance among the neurotypical group (*P *<* *0.001). Supplementary Table 1 provides a summary of the significant effects.

#### Fluent and non-fluent aphasia groups

The distribution of the data is illustrated in Fig. 3. The ANOVA results revealed significant group effects with better performance for the fluent group than the non-fluent group on the quantity of production [*F*(1,44) = 38.11, *P *<* *0.001, partial η^2^ = 0.46], lexical diversity [*F*(1,44) = 48.46, *P *<* *0.001, partial η^2^ = 0.52], informativeness [*F*(1,44) = 35.04, *P *<* *0.001, partial η^2^ = 0.44], and speech rate [*F*(1,44) = 28.15, *P *<* *0.001, partial η^2^ = 0.39]. There were significant main effects of discourse genre for quantity of production [*F*(2,88) = 45.18, *P *<* *0.001, partial η^2^ = 0.51], lexical diversity [*F*(2,88) = 62.84, *P *<* *0.001, partial η^2^ = 0.59], and speech rate [*F*(2,88) = 5.01, *P *=* *0.009, partial η^2^ = 0.1]. A greater number of words were produced during storytelling narrative than picture description and procedural discourse (*P *<* *0.001). For lexical diversity, there was greater diversity during storytelling followed by picture description and then procedural discourse. The differences were significant between storytelling and the other two discourses (*P *<* *0.001), and between picture description and procedural discourse (*P *=* *0.01). Finally, for speech rate, patients spoke significantly faster during procedural discourse compared to both storytelling and picture description (*P *≤* *0.003).

The Group × Discourse interaction effects were significant for quantity of production [*F*(2,188) = 19.19, *P *<* *0.001, partial η^2^ = 0.3] and lexical diversity [*F*(2,188) = 17.48, *P *<* *0.001, partial η^2^ = 0.28]. Both interactions were driven by significantly higher quantity and diversity during storytelling compared to the other two discourses for both groups (*P *≤* *0.01). The differences between picture description and procedural discourse were significant for diversity among the fluent aphasia group only, in favour of picture description (*P *=* *0.01). The differences between the two groups were significant for all three discourses with better performance among the fluent group (*P *<* *0.001). Supplementary Table 1 provides a summary of the significant effects. We repeated the ANOVAs with the addition of lesion size as a covariate. The significant main effects and interaction effects remained the same, and specific lesion size effects (either as main or interaction effects) were not significant in any analysis.

### A unified model of post-stroke language deficits including discourse production

**Figure 4:**
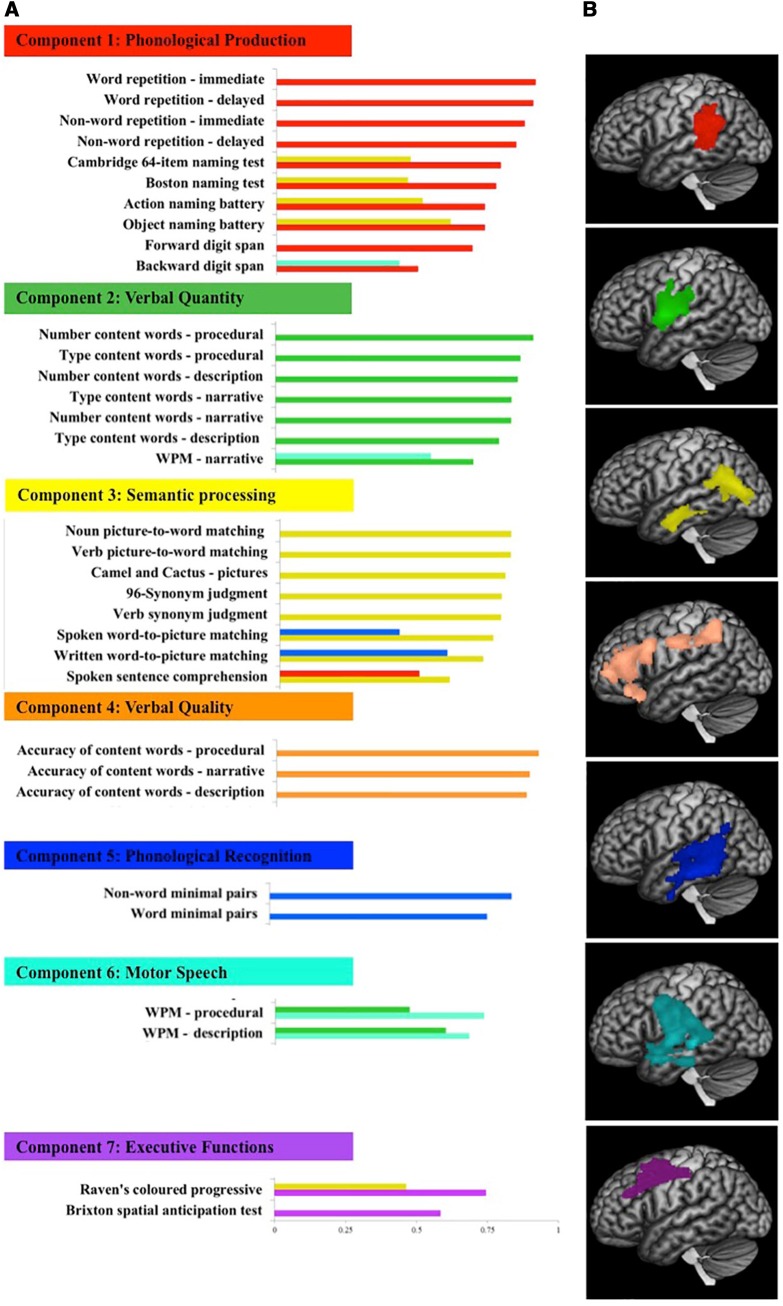
**The fundamental components of post-stroke aphasia and their neural correlates.** (**A**) Loadings of behavioural measures on language and cognitive components extracted from a varimax rotated PCA with loadings > 0.3 (note the loadings of some measures on two components). WPM = words per minute. (**B**) Neural correlates associated with these components identified using VBCM thresholded at *P *<* *0.001 voxel-level and FWE cluster-level corrected at *P *<* *0.05 with demographic variables (age, education, and time post-stroke onset) entered as covariates, except for Component 7, which included lesion volume as covariate (as the cluster associated with this component was not significant without lesion volume correction). Images are at maximum intensity projection.

The PCA generated a seven component solution as indicated by both the eigenvalue cut-off and the cross-validation. This solution accounted for 85.05% of the variance (Fig. 4). Naming and repetition tests (e.g. BNT, OANB, and word/non-word repetition) had the largest loadings on the first component (21.58% variance explained), which was interpreted as ‘phonological production’. The second component (19.31% variance explained) had highest loadings from count measures (content word counts and type counts) from all three discourse samples, and hence was interpreted as ‘verbal quantity’. The third component (19.12% variance explained) loaded with comprehension tests that probe semantic knowledge (e.g. picture-to-word matching and synonym judgement tests) and thus this component was interpreted as ‘semantic processing’. The fourth component (7.87% variance explained) loaded with informativeness from the three discourse samples, and thus was interpreted as ‘verbal quality’. The fifth component (6.82% variance explained) loaded with two phonemic discrimination tests (word and non-word minimal pairs), and hence was interpreted as ‘phonological recognition’. The sixth component (6.16% variance explained) composed of WPM from all three discourse samples, therefore it was interpreted as ‘motor speech’. The final component (4.19% variance explained) was interpreted as ‘executive function’ as it loaded with Brixton spatial anticipation and Raven’s progressive matrices.

### Neural correlates of connected speech alongside other language and cognitive domains

For clarity, results without lesion volume as a covariate are discussed first. Significant clusters were identified for all components except executive function. The neural correlates for the components related to connected speech mainly cover left frontal regions and their underlying white matter tracts. Anatomical labels for brain regions were obtained using the Harvard-Oxford atlas (Desikan *et al.*, 2006), whereas anatomical labs for white matter tracts were described using the NatBrainLab white matter atlas based on diffusion tensor tractography (Catani *et al.*, 2012) in MNI space. The ‘verbal quantity’ component showed neural correlates in the pre/post-central gyri, inferior frontal gyrus (pars opercularis), frontal and central opercular cortices, and the underlying white matter tracts corresponding to the anterior and long segments of the arcuate fasciculus, and the frontal aslant tract. On the other hand, the ‘verbal quality’ component showed neural correlates across left frontal regions, including the inferior frontal gyrus (pars opercularis and pars triangularis), middle frontal gyrus, orbito-frontal cortex, frontal pole, and extended regions including the left superior parietal lobule, superior lateral occipital cortex, and anterior cingulate gyrus. The ‘motor speech’ component showed neural correlates in the pre/post-central gyri, central opercular cortex, orbito-frontal cortex, and deep temporal tissues including Heschl’s gyrus and planum polare, in addition to the fornix and cingulum. Interestingly, although these behavioural components are orthogonal, they share some degree of anatomical overlap. For example, ‘verbal quantity and quality’ overlapped across the pars opercularis, post-central gyrus, whereas ‘verbal quantity and motor speech’ overlapped in the precentral gyrus.

In relation to the neural correlates for the remaining fundamental components, left temporal regions comprising the anterior and posterior inferior and middle temporal gyri, and extended regions involving the planum polare and superior lateral occipital cortex along with tracts corresponding to the inferior longitudinal fasciculus were identified with both ‘phonological recognition’ and ‘semantic processing’ components. Furthermore, the ‘semantic processing’ component correlated with the inferior lateral occipital cortex, anterior temporal fusiform cortex, and white matter tracts corresponding to the uncinate, cingulum, and inferior fronto-occipital fasciculus. On the other hand, parietal regions involving the angular gyrus and posterior supramarginal gyrus, and tracts corresponding to the posterior segment of the arcuate fasciculus correlated with both ‘phonological recognition’ and ‘phonological production’ components. ‘Phonological recognition’ further correlated with Heschl’s gyrus and planum temporale. ‘Phonological production’ also related to the parietal operculum cortex, and tracts corresponding to the anterior and long segments of the arcuate fasciculus, the inferior longitudinal fasciculus and the cortico-spinal tract. The results are illustrated in Fig. 4 and significant clusters and peak MNI coordinates are listed in Table 2.

**Table 2:** Significant clusters and peak MNI coordinates related to language and cognitive components extracted from a varimax rotated PCA

Principal components	**Location** ^a^	Cluster size (number of voxels)		MNI coordinate		
			*Z*-score	*x*	*y*	*z*
Component 1: Phonological production	Corticospinal tract	1889	4.14	−28	−46	30
	Long segment of arcuate fasciculus		3.95	−30	−34	22
	Parietal opercular cortex		3.88	−32	−36	22
	Posterior segment of arcuate fasciculus		3.84	−40	−44	12
	Angular gyrus		3.62	−34	−54	24
	Anterior supramarginal gyrus		3.55	−60	−40	42
	Posterior supramarginal gyrus		3.48	−50	−46	40
	Inferior longitudinal fasciculus		3.47	−34	−46	0
Component 2: Verbal quantity	Precentral gyrus	2280	4.67	−60	0	8
	Central opercular cortex		4.20	−52	−16	12
	Frontal aslant tract		3.98	−54	3	6
	Frontal opercular cortex		3.91	−48	8	2
	Post-central gyrus		3.47	−54	−14	26
	Long segment of arcuate fasciculus		3.40	−47	−3	21
	Inferior frontal gyrus (pars opercularis)		3.25	−55	8	0
	Anterior segment of arcuate fasciculus		3.02	−44	−14	20
Component 3: Semantic processing	Inferior lateral occipital cortex	1255	4.45	−30	−76	10
	Cingulum		4.42	−10	−56	28
	Superior lateral occipital cortex		3.95	−26	−64	24
	Inferior fronto-occipital fasciculus		3.27	−35	−21	−10
	Posterior middle temporal gyrus		3.15	−40	−58	10
	Anterior middle temporal gyrus	595	3.92	−50	−8	−24
	Inferior longitudinal fasciculus		3.91	−38	−10	−20
	Anterior inferior temporal gyrus		3.85	−44	−10	−32
	Posterior inferior temporal gyrus		3.70	−46	−26	−18
	Anterior temporal fusiform cortex		3.34	−38	−6	−26
	Uncinate		3.25	−37	−5	−19
	Planum polare		3.05	−41	−3	−21
Component 4: Verbal quality	Middle frontal gyrus	2237	3.71	−34	16	32
	Frontal pole		3.54	−16	54	4
	Orbito-frontal cortex		3.54	−34	35	−16
	Anterior cingulate gyrus		2.91	−6	30	6
	Superior lateral occipital cortex	1087	3.98	−20	−50	46
	Superior parietal lobule		3.41	−24	−48	54
	Inferior frontal gyrus (pars opercularis)		2.79	−50	18	17
Component 5: Phonological recognition	Posterior inferior temporal gyrus	6916	4.99	−50	−38	−16
	Posterior middle temporal gyrus		4.89	−58	−26	−18
	Planum temporale		4.07	−60	−28	8
	Angular gyrus		4.04	−52	−52	14
	Anterior inferior temporal gyrus		3.86	−42	−8	−40
	Posterior supramarginal gyrus		3.86	−54	−45	14
	Superior lateral occipital cortex		3.64	−28	−78	22
	Anterior middle temporal gyrus		3.62	−52	−6	−24
	Heschl's gyrus		3.40	−46	−18	4
	Posterior segment of arcuate fasciculus		3.32	−48	−49	16
	Posterior superior temporal gyrus		3.14	−68	−10	4
	Planum polare		3.05	−42	−20	−8
	Inferior longitudinal fasciculus		2.98	−48	−18	−19
Component 6: Motor speech	Cingulum	1124	4.88	−18	−30	−4
	Fornix		3.76	−16	−28	18
	Planum polare	1893	4.49	−46	−10	0
	Central opercular cortex		4.10	−58	−14	12
	Post-central gyrus		4.05	−58	−14	26
	Heschl's gyrus		3.99	−50	−18	10
	Precentral gyrus		3.85	−55	−2	20
	Orbito-frontal cortex	420	3.85	−20	6	−22
Component 7: Executive functions	Superior frontal gyrus	2159	5.07	−18	2	48
	Supplementary motor areas		4.83	−14	−4	50
	Frontal aslant tract		4.13	−14	11	50
	Paracingulate gyrus		4.05	−10	30	30
	Middle frontal gyrus		3.68	−34	2	58
	Precentral gyrus		3.28	−34	−6	52

^a^Anatomical labels obtained using Harvard-Oxford atlas (Desikan *et al.*, 2006) and NatBrainLab white matter atlas based on diffusion tensor tractography (Catani *et al.*, 2012) in MNI space.

All results were thresholded at *P *<* *0.001 voxel-level and FWE cluster-level corrected at *P *<* *0.05 with demographic variables (age, education, and time since stroke onset) entered as covariates, except for Component 7, which included lesion volume as covariate (as the cluster associated with this component was not significant without controlling for lesion volume).

None of the seven fundamental components correlate with lesion volume except for the semantic processing component (*r* = −0.54, *P *<* *0.001). On the other hand, the VBCM model with lesion volume correction revealed significant correlations between brain regions and three components: ‘phonological production’, ‘phonological recognition’ and ‘executive functions’, while two components, ‘verbal quantity’ and ‘motor speech’, showed significant clusters at a lenient threshold of *P *=* *0.05 voxel-level and FWE-corrected cluster level at *P *<* *0.01. Overall, the significant clusters corresponded exactly to the earlier results (i.e. without lesion volume correction), except for being smaller in size. The ‘executive function’ component correlated with the left precentral gyrus, superior and middle frontal gyri, supplementary motor areas, paracingulate gyrus, and the frontal aslant tract. These frontal regions showed overlap with the regions identified in association with the connected speech components.

## Discussion

Connected speech production forms the basis of natural communication but it involves a complex interplay between cognitive and linguistic skills. These skills are often assessed using simple picture description tasks, which may miss the true breadth of the mechanisms that underpin more naturalistic forms of connected speech. Furthermore, these skills are often considered in isolation and not alongside other aspects of the language and cognitive processes, which might support at least some of the necessary mechanisms. All of these issues were tackled in the current study of post-stroke aphasia by exploring the production of multiple discourse genres alongside a detailed battery of language and cognitive tests. Four main findings were documented in this study. First, there were quantitative differences related to the amount and diversity of language production probed by different discourse genres. Second, individuals with post-stroke aphasia were poorer than neurotypical adults on all connected speech measures and across all discourse genres, and relatively poorer performance in non-fluent than fluent aphasia patients. Third, connected speech can be supported by at least three fundamental components: the amount of language production, the quality of production related to information accuracy, and motor speech. To date, this provides the most complete model of post-stroke aphasia, which includes seven components: phonological production, phonological recognition, semantic processing, verbal quantity, verbal quality, motor speech and executive functions. Finally, a wide range of brain regions across the left hemisphere supports these aphasia components, with distinct but some overlapping areas. The theoretical and clinical implications of these findings are discussed below.

### Differences across discourse genres

The current findings provided empirical evidence to support the view that the nature of the task places different cognitive and linguistic demands, resulting in quantitative differences in responses to different tasks (Bliss and McCabe, 2006; Fergadiotis and Wright, 2011). Specifically, the findings showed differences in the quantity, diversity and rate of speech in response to different discourse genre. Picture-supported storytelling narrative elicited a higher quantity and lexical diversity of content words than composite picture description and unsupported procedural discourse, albeit at a cost of taking approximately twice as long [mean duration in seconds: (i) storytelling: 128.58 and 190.04; (ii) descriptive discourse: 49.32 and 95.09; and (iii) procedural discourse: 49.89 and 61.95, for neurotypical adults and patients with aphasia, respectively]. Regardless, informativeness does not drop during storytelling narrative, suggesting that participants do utilize this additional time effectively. Strikingly, the pattern was consistent for neurotypical adults and people with aphasia (fluent and non-fluent). Storytelling narratives require identification of participants and their actions, highlighting the events, along with the additional temporal and spatial shifts in chronological order between episodes and thematic relationship between the events (Ulatowska and Olness, 1997). This is likely to impact directly on the amount and diversity of language produced. Overall, this is consistent with previous studies that compared storytelling to picture description in aphasia and neurotypical adults (Fergadiotis and Wright, 2011), and fluent compared to non-fluent aphasia (Wright *et al.*, 2003). The quantity of production and lexical diversity were relatively similar across picture description and procedural discourse in all groups. In contrast, speech rate was slowest during storytelling narrative in all groups, and it was fastest during procedural discourse. This could be because tasks that involve visual stimuli lead to delays in finding specific words for items/events in the pictures, ultimately reducing the speech rate, as procedural discourse was the only discourse in this study that was elicited without pictures. Given that the current study revealed quantitative differences in connected speech in response to different discourse genres, future studies could focus on exploring other measures, such as error types and syntactic and grammatical complexity between different discourse genres.

### Group differences

Patients with aphasia performed lower than neurotypical adults on all measures across all discourse genres. This could not be attributed to a lack of engagement or effort, as patients spent more time on each discourse compared to neurotypical adults (see above). These findings extend on previous small-scale studies that showed reduced lexical diversity in aphasia (Bastiaanse and Jonkers, 1998; Fergadiotis and Wright, 2011), and reduced amount of speech in moderate aphasia (Ulatowska *et al.*, 1983*a*, *b*) compared to neurotypical adults. The current study also showed that although informativeness was at ceiling for neurotypical adults, it was lower in the patient group across all discourse genres. This could reflect the presence of aphasia-related symptoms, such as preservations, repetitions and self-comments, which leads to reduction in information accuracy. Patients with aphasia were slower than the neurotypical adults on all discourse genres, which might reflect the presence of pauses and hesitations. Within the patient group, as one would expect, the fluent patients performed better in the quantity of production and lexical diversity than the non-fluent group across all discourse genres, which has been previously shown for a picture description task (Wright *et al.*, 2003). The current study extended the existing literature by demonstrating the same pattern across storytelling narrative and procedural discourse. These compelling results provide empirical evidence that patients with fluent aphasia are still not as fluent as neurotypical adults (Fig. 2).

### A unified model of post-stroke language deficits

There has been a recent drive in the field to move towards unified models of cognitive behaviour, in the context of attempting to account for multiple domains at once. This has led recent studies to reconceptualize how aphasia is defined using graded deficits along core, orthogonal domains (Kümmerer *et al.*, 2013; Butler *et al.*, 2014; Mirman *et al.*, 2015; Feenaughty *et al.*, 2017; Halai *et al.*, 2017; Lacey *et al.*, 2017; Alyahya *et al.*, 2018*b*). The allure of this approach is that not only does it provide a group level model (in the form of the components themselves), but also an indication of individual differences (in the form of individual variation along each component). Interestingly, the current study provided evidence that connected speech production is supported by three related components instead of just one, in which similar measures from different discourse genres loaded together, forming these components: (i) verbal quantity, related to the amount of language production; (ii) verbal quality, related to producing appropriate and accurate information; and (iii) motor speech component. One would expect the latter to be a component on its own, given that this is a prosodic feature of speech that tends to be prominent in connected speech as opposed to single-word production (Tseng *et al.*, 2005). It is critical to note that these components were identified in the context of a wide range of orthogonal language and cognitive deficits. The full model included: phonological production, semantic processing, phonological recognition, and executive function, all of which have been identified in previous studies on the same or sub-sample of patients (Butler *et al.*, 2014; Halai *et al.*, 2017; Alyahya *et al.*, 2018*b*). Two previous studies have explored connected speech production using a similar data reduction approach (Feenaughty *et al.*, 2017; Halai *et al.*, 2017) but they only found one fluency component. The critical difference might be the use of multiple discourse genres (including more naturalistic forms) in the current study rather than a single picture description task in the previous two studies. Overall, the current large-scale study provides empirical evidence that a combination of verbal quantity, verbal quality and motor speech contributes to the multi-faceted construct of connected speech (Gordon, 1998; Park *et al.*, 2011). It would be interesting for future studies to explore the role of cognitive skills and executive functions during connected speech production, both in neurotypical adults and in patients with aphasia (Robinson *et al.*, 2015).

### Neural correlates

The neural correlates associated with the fundamental components of connected speech production were explored while controlling for the effect of other language processes (e.g. lexical-retrieval and semantic processing) using voxel-wise lesion-symptom mapping. Left frontal and parietal regions were identified in association with the three connected speech components: verbal quantity, verbal quality and motor speech. Specifically, verbal quantity was associated with the left inferior frontal gyrus and pre/post-central gyri, consistent with previous lesion-symptom mapping studies on post-stroke aphasia (Borovsky *et al.*, 2007) and apraxia of speech (Basilakos *et al.*, 2015). There is a large body of evidence to suggest that these dorsal brain regions are important for speech production, as they are related to motor sensory actions and are linked to mapping sounds to production in language models (Hickok and Poeppel, 2004). The cluster associated with verbal quantity also overlays with dorsal connections corresponding to the arcuate fasciculus, aligning with studies that showed that damage to this tract leads to impaired repetition, reduced information and less efficient connected speech (Marchina *et al.*, 2011; Berthier *et al.*, 2012). Also, it has been shown that damage to this tract is a predictor of speech fluency in aphasia during picture description (Fridriksson *et al.*, 2013; Basilakos *et al.*, 2014). The clusters associated with verbal quality involved widespread frontal regions, which have previously been implicated in neuroimaging and neurostimulation studies of higher cognitive functions, including working memory, monitoring, attention and executive semantic control (Gough *et al.*, 2005; Du Boisgueheneuc *et al.*, 2006; Humphreys and Lambon Ralph, 2015). This is consistent for maintaining accurate and high-quality connected speech, as it is contingent on attentional control processes and self-monitoring. A cluster associated with verbal quality also overlaps with the frontal aslant tract, which connects the superior and inferior portions of the frontal lobe (Catani *et al.*, 2013). This tract has been implicated previously with speech fluency in post-stroke aphasia (Basilakos *et al.*, 2014; Halai *et al.*, 2017) and primary progressive aphasia (Catani *et al.*, 2013), and has been shown to disrupt speech (Kinoshita *et al.*, 2015) and lexical retrieval (Sierpowska *et al.*, 2015) during electrical stimulation mapping. On the other hand, two clusters were correlated with motor speech component, one covering the precentral gyrus, and the second one overlays with auditory cortices in the temporal lobe. We know that the former is linked to executing speech mouth movements based on functional neuroimaging experiments (Price *et al.*, 2011), whereas the involvement of auditory regions with this component can be related to online auditory feedback, which is necessary during connected speech production (Sharp *et al.*, 2004). The regions identified in the current study benefit from having controlled for the influence of other language processes, such as phonological production, lexical-retrieval, and executive functions in a unified model, as well as demographic factors. However, it must be acknowledged that white matter tracts described in this study are descriptive, based on the overlap of the significant clusters with a white matter atlas (Catani *et al.*, 2012) rather than directly measured using diffusion-weighted imaging. Future studies could use connectome-based approaches as a direct measure of the role of the identified tracts with connected speech production.

The neural correlates associated with the remaining four components replicated findings from a previous study (for details, see Alyahya *et al.*, 2018*b*). Findings from this study provide converging evidence with previous lesion mapping studies (Fridriksson *et al.*, 2010; Hartwigsen *et al.*, 2010; Schwartz *et al.*, 2012; Kümmerer *et al.*, 2013; Butler *et al.*, 2014; Halai *et al.*, 2017) and prominent models of language processing (Hickok and Poeppel, 2004; Saur *et al.*, 2008; Price, 2012; Kümmerer *et al.*, 2013). In brief, middle and superior temporal regions were related to phonological recognition, whereas middle and ventral anterior temporal regions were involved with semantic processing. Left parietal regions were related to phonological production, which is involved in repetition and phonological retrieval (Fridriksson *et al.*, 2010; Kümmerer *et al.*, 2013). Executive functions were associated with left frontal regions, which have been previously implicated with executive processing (Duncan and Owen, 2000; Garic *et al.*, 2018).

## Conclusion

Novel empirical evidence was obtained for the storytelling narrative as a rich data source that is not available when using picture description or procedural discourse, even when no time limits were placed on responses. Picture-supported storytelling narratives benefit from being a naturalistic mode of communication with reduced memory load. This has clinical implications related to the sensitivity of examination and planning therapeutic interventions, as well as affecting research activities. Given the time it takes to code the connected speech responses, future work should focus on determining ways to make coding features more efficient. The current study also identified three orthogonal core connected speech components (verbal quantity, verbal quality and motor speech) in a unified model of post-stroke language deficits. The full model consists of four additional orthogonal components: phonological production, semantic processing, phonological recognition, and executive functions. These seven components were supported by left frontal, temporal and parietal regions, where some areas overlapped but others were distinct.

## Supplementary Material

awaa074_Supplementart_Data

## Abbreviations

PALPA =: Psycholinguistic Assessments of Language Processing in Aphasia
PCA =: principal component analysis
