# Supplementary material for: A unified model of post-stroke language deficits including discourse production and their neural correlates
Source: Brain. 2020 Apr 24;143(5):1541–54. doi: 10.1093/brain/awaa074 (PMC7241958; doi:10.1093/brain/awaa074)
Supplement: awaa074_Supplementart_Data [file awaa074_supplementart_data.docx]

**A unified model of post-stroke language deficits including discourse production and their neural correlates - Supplementary Materials**

Reem S. W. Alyahya, Ajay D. Halai, Paul Conroy, and Matthew A. Lambon Ralph

## Acquisition and processing of neuroimaging data

High-resolution structural T1-weighted MRI scans were acquired for each participant on a 3 T Philips Achieva scanner (Philips Healthcare, Best, The Netherlands) using an eight-channel SENSE head coil. A T1-weighted inversion recovery sequence with 3D acquisition was utilised, with the following parameters: repetition time = 9.0 ms, echo time = 3.93 ms, acquired voxel size = 1.0 × 1.0 × 1.0 mm^3^, slice thickness = 1 mm, matrix size = 256 × 256, 150 contiguous slices, flip angle = 8, field of view = 256 mm, inversion time = 1150 ms and SENSE acceleration factor 2.5 resulting in a total scan time of 575 seconds.

Participants' structural T1-weighted MRI scans were pre-processed with Statistical Parametric Mapping software (SPM8: Wellcome Trust Centre for Neuroimaging, <http://www.fil.ion.ucl.ac.uk/spm/>) running under MATLAB (2012a). Initially, non-brain tissues from the T1 images were stripped using an optimised brain extraction tool for lesioned brains (OptiBET: Lutkenhoff *et al.*, 2014). The resultant images were then normalised into standard Montreal Neurological Institute (MNI) space using a modified unified segmentation-normalisation procedure optimised for focal brain lesions (Seghier *et al.*, 2008). Structural imaging scans from a healthy age- and education-matched control group (18 males and 4 females; mean age = 69.13 years, SD = 5.85, range = 59–80; and mean education = 13 years, SD =2 .66, range=10–18) were used as a reference to identify lesion/abnormal tissue in the stroke patients (note these were not the same controls as in the neuro-typical behavioural dataset). Structural MRI scans from both groups were entered into the segmentation-normalisation procedure. This procedure combines segmentation, spatial normalisation and bias correction through the inversion of a single unified model, which combines tissue classes (grey and white matter, cerebral spinal fluid (CSF), and an additional tissue class for abnormal voxels), intensity bias and non-linear warping into the same probabilistic models that are assumed to generate individual-specific brain images (details available in Ashburner and Friston, 2005). This procedure essentially detects areas of neural abnormality in an unexpected tissue class, and therefore, identifies missing grey and white matter as well as areas of augmented CSF space. Each patient's lesion was automatically identified using this fully automated method based on fuzzy clustering (Seghier *et al.*, 2008), using a ‘U-threshold’ of 0.5 after consulting an expert neurologist. Images were then smoothed with an 8mm full-width-half-maximum Gaussian kernel, and were then used in the lesion-symptom mapping analyses. The images generated for each patient were individually checked and visually inspected with respect to the original scan and then used to generate a lesion overlap map (Figure 1).

Supplementary Table 1: Significant effects of 2 (group) × 3 (discourse genre: narrative vs. descriptive vs. procedural) mixed ANOVAs

| **Dependent variable**  **Conditions** | **Quantity (content word count)** | **Diversity (content word type count)** | **Quality (informativeness)** | **Speech rate (words-per-minute)** |
| --- | --- | --- | --- | --- |
| **Group (neuro-typical versus aphasia)** | * | * | * | * |
| **Discourse** | * | * | - | * |
| **Group** × **discourse** | * | * | - | - |
|  |  |  |  |  |
| **Group (fluent versus non-fluent aphasia)** | * | * | * | * |
| **Discourse** | * | * | - | * |
| **Group** × **discourse** | * | * | - | - |
| - Not significant, * Significant effect at p < 0.01 | | | | |

**Refrences:**

Ashburner J, Friston KJ. Unified segmentation. NeuroImage 2005; 26: 839-51.

Lutkenhoff ES, Rosenberg M, Chiang J, Zhang K, Pickard JD, Owen AM*, et al.* Optimized brain extraction for pathological brains (optiBET). PLoS ONE 2014; 9: e115551.

Seghier ML, Ramlackhansingh A, Crinion JT, Leff AP, Price C. Lesion identification using unified segmentation-normalisation models and fuzzy clustering. NeuroImage 2008; 41: 1253-66.
